# Association between infections and frailty: a systematic review protocol

**DOI:** 10.1136/bmjopen-2026-117882

**Published:** 2026-09-09

**Authors:** Aleixo P Brunetti, Mohammed Baothman, Kwabena Asare, Kathryn E. Mansfield, Charlotte Warren-Gash

**Affiliations:** 1 Non-Communicable Disease Epidemiology, London School of Hygiene & Tropical Medicine, London, UK; 2 University of Lincoln, Lincoln, UK

**Keywords:** Frailty, Infection control, Longitudinal studies, Frail Elderly, Aging

## Abstract

**Abstract:**

**Introduction:**

Frailty is a multidimensional syndrome of reduced physiological reserve and heightened vulnerability to stressors. Frailty is common among older adults and linked to adverse outcomes including disability, hospitalisation and death. Infections are common in older populations and remain a major cause of morbidity and death. Although it has been suggested that infections influence frailty development and progression (via inflammatory and functional mechanisms), evidence from longitudinal studies is inconsistent. Therefore, we aim to assess longitudinal associations between infections and frailty in adults.

**Methods and analysis:**

We will conduct a systematic review of observational cohort and case-control studies examining associations between infections and frailty in adults. We will search MEDLINE, Embase and Global Health from 2001 to February 2026 without language restrictions. Two reviewers will independently screen titles and abstracts and subsequently assess full texts for eligibility. Data will be extracted using a standardised data extraction form. Risk of bias will be assessed using a customised domain-based tool based on the Risk Of Bias In Non-randomised Studies of Exposures (ROBINS-E) framework. We will initially narratively synthesise our findings. Where studies are sufficiently homogeneous, we will undertake random effects meta-analysis. We will assess the certainty of the evidence using the Grading of Recommendations Assessment, Development and Evaluation (GRADE) approach.

**Ethics and dissemination:**

Ethical approval is not required for a systematic review. Results of the review will be published in a peer-reviewed journal and disseminated through conferences.

**PROSPERO registration number:**

CRD420261299590.

**STRENGTHS AND LIMITATIONS OF THIS STUDY**

- Our systematic review will critically synthesise longitudinal evidence on how infections are associated with subsequent changes in frailty status in adults.
- Our review protocol follows the Preferred Reporting Items for Systematic Review and Meta-Analysis Protocols (PRISMA-P) guidelines, uses a predefined eligibility framework and is prospectively registered in the International Prospective Register of Systematic Reviews (PROSPERO CRD420261299590).
- We will increase the likelihood of identifying all relevant studies through a comprehensive search strategy across multiple databases with no limitation to specific languages.
- Our ability to infer causality from any pooled estimates may be limited, as it is likely our review will synthesise observational studies that differ in design (cohort and case-control) and in the confounders adjusted for, meaning that there may be reasons, other than causality (eg, residual confounding), to explain any observed associations. However, results from methodologically robust studies can still offer meaningful insights into potential causality.
- Studies of incident frailty and transitions between frailty states often have imprecise timing of frailty onset due to infrequent frailty assessments, leading to potential interval-censoring and misclassification, which may bias the effect estimates.

## Introduction

 Frailty is a complex clinical syndrome characterised by reduced physiological reserve and increased vulnerability to health stressors.^1 2^ Frailty commonly affects older adults, and it is associated with a range of adverse outcomes, including reduced quality of life and increased disability and mortality.^2 3^ In parallel, infections are common in ageing populations and represent a major cause of mortality and morbidity.^4^

Frailty is common, with an estimated prevalence of 18% (95% CI 15% to 21%) among Europeans aged 50.^5^ Frailty prevalence varies by factors including age, underlying health conditions, socioeconomic status and access to healthcare.^6^ Infections not only occur more frequently in those with frailty and disability, but are also more likely to progress to life-threatening complications.^6 7^

Emerging evidence suggests infections may play a direct role in frailty onset and progression.^2^ Acute infections are known to induce systemic inflammation, which can lead to muscle catabolism and functional decline, thereby potentially accelerating frailty trajectories.^8 9^ Chronic infections (eg, those caused by cytomegalovirus (CMV) and HIV) are associated with persistent immune activation, sarcopenia and multimorbidity, which are all likely to exacerbate frailty.^9–12^ Inflammatory biomarkers such as interleukin-6 (IL-6) and other pro-inflammatory cytokines are elevated in both frailty and chronic infection, supporting the hypothesis of shared inflammatory pathways.^10 13 14^ Factors like hospitalisation for infections, prolonged bed rest and iatrogenic complications may further exacerbate frailty in already vulnerable individuals.^15^

Evidence investigating the link between infections and frailty is mixed, with some studies reporting evidence of associations between specific infections and frailty^11 12^ and others finding no clear association.^16^ These discrepancies may reflect differences in study design, residual confounding, small sample sizes or variation in how infections and frailty are measured. In addition, the prevalence of frailty and incidence of infections vary widely across populations and settings; where event rates are low or where the expected effect sizes are small, much larger sample sizes than are typically available are needed to detect associations reliably, reducing statistical power and increasing the influence of bias. Evidence also varies by infection type and severity and analytical approach. It is unclear whether particular infections are more strongly linked to frailty progression or whether associations differ by age, sex or comorbidities.

We will therefore conduct a systematic review to synthesise longitudinal evidence on how infections influence frailty. Bringing together the existing literature will clarify whether there is consistent evidence that specific infections or patterns of infection are associated with worsening frailty, identify gaps in current knowledge and potentially support the development of targeted prevention and clinical management strategies for older adults.

## Aim and objectives

The aim of our systematic review is to provide an up-to-date appraisal of the evidence on the association between infections and frailty in adults and, if a meta-analysis proves possible, synthesise pooled estimates for the association.

Our objectives are to:

1. Synthesise longitudinal evidence on whether infections are associated with subsequent frailty (including frailty incidence, progression to more severe frailty states and changes in frailty level over time).
2. Where included studies allow, explore how associations between infections and frailty differ according to infection characteristics (type, timing, severity, cumulative burden) and across broader sources of heterogeneity, including age, sex, frailty definition, study design and settings.

We hypothesise that the existing evidence will show that infections are associated with a higher subsequent risk of frailty onset and faster frailty progression, and that the strength of these associations will vary according to infection characteristics (type, timing, severity and cumulative burden) and across other sources of heterogeneity, including age, sex, frailty definition, study design and setting.

## Methods

### Protocol and registration

This protocol is reported in accordance with the *Preferred Reporting Items for Systematic Review and Meta-Analysis Protocols* (PRISMA-P) guidelines.^17^ The PRISMA-P checklist is provided as online supplemental table 1. Our completed systematic review will be reported in line with the *Preferred Reporting Items for Systematic Review and Meta-Analysis* (PRISMA) 2020 statement.^18^

This protocol has been registered at the International Prospective Register of Systematic Reviews (PROSPERO) (reference: CRD420261299590). Any changes made to this protocol will be recorded in PROSPERO and reported in the final manuscript.

### Eligibility criteria

We used the PECOS (Population, Exposure, Comparator, Outcomes and Study design) framework to formulate our review’s research question and eligibility criteria.^19^

#### Population

Studies including adults aged 18 years or older will be eligible. We will include studies with study populations including individuals under 18 years of age, if results for adults can be clearly identified. We will not apply any restrictions based on sex, ethnicity or geographical location.

#### Exposure

Eligible studies must assess infection exposures (eg, any infections or specific infectious diseases such as respiratory infections, urinary tract infections, HIV or cytomegalovirus) or infection-related exposures (including measures of infection burden such as infection frequency, severity, duration, hospitalisation for infection or cumulative exposure over time). Infection exposure may be ascertained through (but is not limited to): clinical or administrative records, laboratory-confirmed diagnoses, disease registries, healthcare utilisation data or self-reported and physician-diagnosed infection.

We will exclude studies that focus exclusively on post-infectious syndromes or sequelae, including post-COVID syndrome (also referred to as long COVID-19 or post-acute sequelae of SARS-CoV-2 infection), because these conditions do not necessarily indicate the presence of active or chronic infection.

#### Comparator

We will only include studies with an appropriate comparator population. Comparator groups may include individuals without the infection exposure or groups differing in infection severity, frequency or cumulative infection burden (eg, mild vs severe infections or infections requiring hospitalisation vs hospitalisation for non-infectious elective surgical procedures), where such contrasts allow assessment of the relationship between infection exposure and frailty outcomes.

#### Outcomes

Studies must report a frailty-related outcome measured using any frailty assessment method, including the frailty phenotype,^3^ frailty index^20^ or other structured frailty scales.

The primary outcome will be frailty incidence. We acknowledge that capturing the onset of frailty presents methodological challenges, as frailty exists on a continuum and is typically assessed at discrete time points, which may result in imprecise estimation of the timing of its onset. Our secondary outcome will be frailty progression (including transitions between categorical frailty states and longitudinal change in continuous frailty score).

#### Study design

We will include longitudinal observational studies (ie, cohort and case-control studies (including nested case-control designs)) examining the association between infections or infection-related exposures and frailty outcomes.

We will exclude cross-sectional studies, ecological studies, interventional studies, case reports or case series, review articles, editorials and commentaries. However, we will review the studies included in any relevant review articles for potential inclusion in our study.

#### Search strategy

We will conduct a comprehensive literature search to identify relevant published studies examining the association between infections and frailty. The search strategy has been developed iteratively by the review team, informed by preliminary searches and refinement of keywords and index terms identified from relevant studies. The search strategy was subsequently reviewed by a specialist librarian.

We will search the following electronic databases for relevant articles from 2001 to February 2026: MEDLINE, Global Health and Embase (via Ovid). These databases were selected because they provide broad and complementary coverage of the biomedical, public health and global health literature, including extensive indexing of observational epidemiological studies on infections and frailty.^19 21^ Other databases will not be included due to substantial overlap in content with these core sources or have more limited relevance to the clinical and epidemiological focus of this review.^21^

Publication dates will be restricted to studies published from 2001 onwards, corresponding to the year in which the frailty phenotype was first formally described.^3^ We will search medical subject headings and free text (in titles, abstracts and keywords) for synonyms relating to two key concepts: (1) frailty (eg, ‘frailty’, ‘frailty index’, ‘frailty phenotype’); and (2) infection (eg, ‘infection’, ‘infectious disease’, ‘viral’, ‘bacterial’, ‘chronic infection’). We will not apply any language restrictions, and translations will be sought where necessary. Our search string was first developed for MEDLINE and subsequently adapted for the other databases online supplemental table 2.

### Study records

#### Data management

All records identified through the literature search will be collated in EndNote 2025 (Clarivate) (reference management software), and duplicate records will be removed prior to screening.^22^ The de-duplicated records will then be exported to Rayyan (a web-based tool designed to support the screening and management of systematic reviews) for screening, where any remaining duplicates will be identified manually and removed.^23^ Screening will be conducted in Rayyan using blinded reviewer settings.

#### Selection process

Study selection will be performed in two stages: (1) title and abstract screening and (2) full-text review. Two reviewers (APB and MB) will independently screen all records at both stages against the predefined eligibility criteria. Any disagreements between reviewers at either stage will be resolved through discussion, with a third reviewer (KM) consulted if consensus cannot be reached. To minimise potential conflicts of interest, where a member of the review team has authored a paper identified by the search, that individual will not participate in decisions regarding the eligibility of the study and an alternative reviewer will be consulted.

The number of records identified, screened, assessed for eligibility and included in the review, along with reasons for exclusion at the full-text stage, will be documented and presented using a PRISMA flow diagram.

### Risk of bias assessment

We will assess risk of bias of all included studies using a customised domain-based tool based on the Cochrane Risk Of Bias In Non-randomised Studies of Exposures (ROBINS-E) framework.^24^ The customised tool will aim to capture key sources of bias relevant to observational studies of infection and frailty in the following domains: (1) confounding, (2) participant selection, (3) exposure misclassification, (4) outcome misclassification, (5) missing data, (6) reverse causation and (7) generalisability and study power. For each study, each domain will be rated as low-, moderate- or high-risk of bias, with concise justifications recorded for transparency.

Two reviewers will independently assess all included studies for risk of bias. Any disagreements will be resolved through discussion, with a third reviewer available if consensus cannot be reached. Studies will not be excluded based on risk of bias alone. Assessments of methodological quality and certainty of evidence will be used to inform the interpretation of findings and to support sensitivity analyses, where appropriate.

### Confidence in cumulative evidence

We will evaluate the overall certainty of the body of evidence using the Grading of Recommendations Assessment, Development and Evaluation (GRADE) approach.^25^ For each outcome (ie, frailty incidence or frailty progression), the certainty of evidence will be classified as high, moderate, low or very low. In accordance with GRADE guidelines, evidence may be upgraded where findings demonstrate a substantial effect size or indicate a dose-response relationship. Conversely, the certainty of evidence may be downgraded in the presence of methodological limitations, imprecise estimates, inconsistent findings across studies or suspected publication bias. We will present the GRADE evaluations in a ‘Summary of Findings’ table.

### Data extraction

We will extract data from all included studies using a standardised data extraction form developed by the review team. The data extraction form will be pilot tested on a subset of included studies and refined as necessary to ensure clarity, completeness and consistency. To assess consistency between reviewers, two reviewers (APB and MB) will independently extract data from either five included studies or a 10% sample of included studies, whichever is larger. Data from the remaining studies will be extracted by a single reviewer (APB). If disagreement between reviewers is identified in more than 10% of extracted fields, an additional 10% of included studies, or five studies, whichever is greater, will be extracted by both reviewers independently, and the extraction form refined before single-reviewer extraction resumes.

Where available, we will extract information for the following: study characteristics (authors, year of publication, study title, aim, study design, country and setting); participant characteristics (sample size, age (mean and SD or median and IQR), sex distribution and any other relevant characteristics); infection exposure definition (eg, type of infection, pathogen, timing, severity, frequency, cumulative infection burden and measurement method); frailty assessment (frailty definition or tool used, timing of assessment and outcome type including incidence or progression); and outcome measure (effect estimates, measures of uncertainty, follow-up duration and covariates adjusted for); and information on missing data.

## Data synthesis

### Narrative synthesis

We will undertake a structured narrative synthesis to summarise findings from all included studies. We will describe and tabulate the results of the included studies according to key characteristics, including study design (cohort, case-control), infection exposure definition, frailty assessment method and whether frailty outcome is assessed via incidence or progression. Associations between infection-related exposures and frailty outcomes will be compared across studies, taking risk of bias assessment into account.

### Meta-analysis

We will consider quantitative synthesis if at least three studies are identified that directly examine the association between infections or infection-related exposures and frailty outcomes, and if these studies are sufficiently homogeneous in terms of study design, study population and definitions of infection exposure and frailty outcome.^19^ Decisions regarding data pooling will be informed by an assessment of clinical and methodological comparability. Pooled estimates will be based on adjusted effect estimates, where available.

For categorical frailty outcomes, we will extract or compute ORs, risk ratios or hazard ratios (effect estimate appropriate to specific study) with 95% CIs. For continuous frailty outcomes, depending on the results presented in the specific study, we will extract: mean differences, standardised mean differences or regression coefficients. When different frailty scales are used, we will convert continuous effect estimates to standardised mean differences to enable pooling on a common scale. Regression coefficients will be synthesised using the generic inverse variance method where appropriate.^26^

Included studies are likely to differ in follow-up duration and in the number and timing of frailty assessments. For studies with repeated frailty assessment, we will tabulate follow-up length, number of time points and time metric (ie, whether change is modelled against time-since-baseline or age) for every included study where these apply (recognising that case-control and other non-longitudinal designs may not report follow-up duration or repeated frailty assessments). We will group studies with follow-up data by duration for pre-specified subgroup analysis. Where studies report multiple frailty measurement time points, the primary meta-analysis will use each study’s primary reported estimate (in most cases the longest available follow-up), irrespective of differences in total follow-up duration between studies. Results at shorter time points will be described narratively and, where a sufficient number of studies report estimates at comparable intervals (eg, 1 year or 2 years) and are judged sufficiently homogeneous, we will pool these time-point-specific estimates in secondary analyses.^5^ Where time-point heterogeneity precludes meaningful pooling, we will restrict quantitative synthesis to comparable subsets and describe the remainder narratively.

We will assess statistical heterogeneity using the Cochran Q test, with p<0.05 taken as indicative of heterogeneity and quantified using the I² statistic.^27^ I² values of approximately 25%, 50% and 75% will be interpreted as reflecting low, moderate and substantial heterogeneity, respectively.^27^ Given the anticipated heterogeneity across studies, we will use a random-effects meta-analysis.^28^ The extent of heterogeneity will be considered when interpreting pooled estimates.

Where the included studies permit, we will explore potential sources of heterogeneity through subgroup analyses, including by age, sex and type of infection exposure.^29^ If possible, we will examine the robustness of pooled results through sensitivity analyses, including leave-one-out analyses and the exclusion of studies at higher risk of bias.^30^

### Meta-bias

If possible (ie, if there are multiple studies estimating the same type of effect estimates), we will assess small-study effects and publication bias through visual inspection of funnel plots. In addition, we will use Egger’s regression test to formally assess funnel plot asymmetry. A p value <0.05 will be considered indicative of potential asymmetry and publication bias.^26^

## Discussion

To our knowledge, no recent systematic review has synthesised longitudinal evidence on the association between infections and subsequent frailty across pathogens in adults. Existing reviews have addressed cross-sectional studies of herpesvirus seropositivity, where cytomegalovirus but no other herpesvirus was associated with frailty^31^; prevalence of frailty in people with HIV, without an uninfected comparator^32^; or the reverse direction of association (frailty as a risk factor for infection outcomes). This review will therefore address an important gap in the evidence base.

We anticipate several challenges in synthesising the identified literature. Included studies are likely to differ in infection exposures (from single pathogens to broad measures of infection burden), frailty measurement instruments, populations, follow-up duration and the timing of frailty assessment relative to infection. Residual confounding is likely to remain even in well-adjusted analyses and combining different observational designs will introduce further heterogeneity in how confounders are handled. To manage these challenges, we will use structured narrative synthesis as the default and reserve meta-analysis for subsets of studies sufficiently homogeneous to permit pooling. Data management will follow a pre-specified extraction form piloted on a subset of included studies.

## Ethics and dissemination

Ethical approval is not required for this systematic review, as it will synthesise data from previously published studies and will not involve the collection of primary data from human participants.

The findings of this review will be disseminated through peer-reviewed journals, presentations at relevant scientific conferences and a linked post on the institutional website. Where appropriate, we may also present the findings in webinar format to enable ongoing online access for international research and clinical audiences and share plain-language summaries through institutional channels including the LSHTM Brain Health Group website and academic social media. The results are expected to be of interest to clinicians, researchers and policymakers involved in ageing, frailty and infectious disease research, and may help inform future research and clinical practice.

## Supplementary material

10.1136/bmjopen-2026-117882 online supplemental file 1
