# Supplementary material for: Association between infections and frailty: a systematic review protocol
Source: BMJ Open. 2026 Sep 9;16(9):e117882. doi: 10.1136/bmjopen-2026-117882 (PMC13560988; doi:10.1136/bmjopen-2026-117882)
Supplement: Supplementary file 1 [file bmjopen-2026-117882.reviewer_comments.pdf]

PEER REVIEW HISTORY

BMJ Open publishes all reviews undertaken for accepted manuscripts. Reviewers are asked to complete a checklist review form and are provided with free text boxes to elaborate on their assessment. These free text comments are reproduced below.

ARTICLE DETAILS

Title (Provisional)

The association between infections and frailty: a systematic review protocol

Authors

Brunetti, Aleixo P; Baothman, Mohammed; Asare, Kwabena; Mansfield, Kathryn; Warren-Gash, Charlotte

---

VERSION 1 - REVIEW

**Reviewer**	**1**
**Name**	**Cook, Deborah**
**Affiliation**	**McMaster University**
**Date**	**09-Jun-2026**
**COI**	**I am interested in frailty and embarking on a study to examine whether frailty influences risk of infection (the opposite direction of association as this article).**

This is a well written protocol to assess longitudinal associations between infections and frailty in adults.

The abstract is excellent.

Under Ethics and dissemination, please consider more venues and strategies than in a peer-reviewed journal and conference presentation.

Under the Strengths and Limitations, can you consider adding the concept of confounding due to inclusion of study designs in this review?

The Introduction is well written. To this sentence, “These discrepancies may reflect differences in study design, residual confounding, small sample sizes, or variation in how infections and frailty are measured. “ can you acknowledge the issue of the prevalence of frailty and infection or incidence of frailty and infection. The lower the event rate, all else being equal, the more challenging it is tolt to identify associations, and potentially the higher the risk of bias.

The registration is duly noted.

Would you consider some hypotheses for this retrospective exercise?

Acknowledging that in a protocol for a systematic review, it is difficult, if not unwise, to be too declarative about plans, authors have done a good job identifying their planned approaches.

In the Methods, would you consider referencing the different definitions proposed, as in: Studies must report a frailty-related outcome measured using any frailty assessment method, including the frailty phenotype, frailty index, or other structured frailty scales.

The Methods are comprehensive and even include a statement about conflict of interest for articles coauthored by one of the reviewers.

In the analysis section, what are some strategies for handling the different time points?

For data extraction, you state: "To assess consistency between reviewers, two reviewers will independently extract data from either five included studies or a 10% sample of included studies, whichever is larger. Data from the remaining studies will be extracted by a single reviewer." Can you add a contingency threshold of what degree of disagreement will lead to more duplicate reviews?

A brief Discussion section would be welcome. Is a brief synthesis of the last systematic review on this topic worth citing, or a comment outlining that there is no such publication? The literature could be very disparate. What challenges in synthesis are anticipated (they are mentioned throughout but that could be consolidated here), and what data management strategies will be considered (or are anticipated)?

The publication checklist is appreciated.

This will be a valuable contribution to the literature.

VERSION 1 - AUTHOR RESPONSE

Response to Reviewer 1

COMMENT 1.1 Under Ethics and dissemination, please consider more venues and strategies than in a peer-reviewed journal and conference presentation.

RESPONSE 1.1

Thank you for this suggestion. We have expanded the Ethics and dissemination section to include webinar presentations, plain-language summaries through institutional channels, and academic social media, alongside our original commitments to peer-reviewed publication and conference presentations.

MANUSCRIPT CHANGES 1.1

Location: Ethics and dissemination p 12, lines 347-351

The findings of this review will be disseminated through peer-reviewed journals, presentations at relevant scientific conferences, and a linked post on the institutional website. Where appropriate, we may also present the findings in webinar format to enable ongoing online access for international research and clinical audiences and share plain-language summaries through institutional channels including the LSHTM Brain Health Group website and academic social media.

COMMENT 1.2 Under the Strengths and Limitations, can you consider adding the concept of confounding due to inclusion of study designs in this review?

RESPONSE 1.2

We agree this is an important limitation. To comply with the journal's requirement of a maximum of five bullet points in the Strengths and Limitations box, we have incorporated this point into the existing bullet on causal inference from observational studies, which addresses the same underlying issue of confounding across differing study designs.

MANUSCRIPT CHANGES 1.2

Location: Strengths and limitations of this study p 2, lines 49-54

- Our ability to infer causality from any pooled estimates may be limited, as it is likely our review will synthesise observational studies that differ in design (cohort and case-control) and in the confounders adjusted for, meaning that there may be reasons, other than causality (e.g., residual confounding), to explain any observed associations. However, results from methodologically robust studies can still offer meaningful insights into potential causality.

COMMENT 1.3 The Introduction is well written. To this sentence, "These discrepancies may reflect differences in study design, residual confounding, small sample sizes, or variation in how infections and frailty are measured. " can you acknowledge the issue of the prevalence of frailty and infection or incidence of frailty and infection. The lower the event rate, all else being equal, the more

challenging it is tolt to identify associations, and potentially the higher the risk of bias.

RESPONSE 1.3

We appreciate this comment. This is a useful point that was not previously captured in the paragraph on inconsistency across the existing evidence. We have added a sentence to the Introduction to address it.

MANUSCRIPT CHANGES 1.1

Location: Introduction p 3, lines 85-89

In addition, the prevalence of frailty and incidence of infections vary widely across populations and settings; where event rates are low, or where the expected effect sizes are small, much larger sample sizes than are typically available are needed to detect associations reliably, reducing statistical power and increasing the influence of bias.

COMMENT 1.4 Would you consider some hypotheses for this retrospective exercise?

RESPONSE 1.4

We have added a hypothesis statement at the end of the Aim and Objectives section.

MANUSCRIPT CHANGES 1.4

Location: Aim and Objectives p 4, lines 111-115

We hypothesise that the existing evidence will show that infections are associated with a higher subsequent risk of frailty onset and faster frailty progression, and that the strength of these associations will vary according to infection characteristics (type, timing, severity, and cumulative burden) and across other sources of heterogeneity, including age, sex, frailty definition, study design, and setting.

COMMENT 1.5 In the Methods, would you consider referencing the different definitions proposed, as in: Studies must report a frailty-related outcome measured using any frailty assessment method, including the frailty phenotype, frailty index, or other structured frailty scales.

RESPONSE 1.5

We have added references for the frailty phenotype (Fried et al.) and the frailty index (Rockwood and Mitnitski) at the point where these methods are first named in the Methods section.

MANUSCRIPT CHANGES 1.5

Location: Outcomes p 6, line 158

Studies must report a frailty-related outcome measured using any frailty assessment method, including the frailty phenotype,[3] frailty index,[20] or other structured frailty scales.

References:

3. Fried LP, Tangen CM, Walston J, et al. Frailty in Older Adults: Evidence for a Phenotype. *The Journals of Gerontology: Series A* 2001;56(3):M146–M57. doi: 10.1093/gerona/56.3.M146

20. Rockwood K, Mitnitski A. Frailty in Relation to the Accumulation of Deficits. *The Journals of Gerontology: Series A* 2007;62(7):722–27. doi: 10.1093/gerona/62.7.722

COMMENT 1.6 In the analysis section, what are some strategies for handling the different time points?

RESPONSE 1.6

This is a helpful prompt. We have added a paragraph to the meta-analysis section describing our strategies for handling differences in follow-up duration, and in the number and timing of frailty assessments across included studies.

MANUSCRIPT CHANGES 1.6

Location: Meta-analysis p 10-11, lines 289-304

Included studies are likely to differ in follow-up duration and in the number and timing of frailty assessments. For studies with repeated frailty assessment, we will tabulate follow-up length, number of time points, and time metric (i.e., whether change is modelled against time-since-baseline or age) for every included study where these apply (recognising that case-control and other non-longitudinal designs may not report follow-up duration or repeated frailty assessments). We will group studies with follow-up data by duration for pre-specified subgroup analysis. Where studies report multiple frailty measurement time points, the primary meta-analysis will use each study's primary reported estimate (in most cases the longest available follow-up), irrespective of differences in total follow-up duration between studies. Results at shorter time points will be described narratively and, where a sufficient number of studies report estimates at comparable intervals (for example, one year or two years) and are judged sufficiently homogeneous, we will pool these time-point-specific estimates in secondary analyses.[27] Where time-point heterogeneity precludes meaningful pooling, we will restrict quantitative synthesis to comparable subsets and describe the remainder narratively.

Reference:

27. Welstead M, Jenkins ND, Russ TC, et al. A Systematic Review of Frailty Trajectories: Their Shape and Influencing Factors. *The Gerontologist* 2021;61(8):e463–e75. doi: 10.1093/geront/gnaa061

COMMENT 1.7 For data extraction, you state: "To assess consistency between reviewers, two reviewers will independently extract data from either five included studies or a 10% sample of included studies, whichever is larger. Data from the remaining studies will be extracted by a single reviewer." Can you add a contingency threshold of what degree of disagreement will lead to more duplicate reviews?

RESPONSE 1.7

Thank you for your suggestion. We have added a pre-specified contingency threshold of 10% field-level disagreement to the Data Extraction section, at which point an additional 10% of included studies (or five, whichever is greater) will be dual-extracted. We chose 10% because it is high enough to distinguish genuine inconsistency in extraction from isolated minor discrepancies, but low enough to trigger corrective

action before problems propagate through the remaining single-reviewer extraction. In addition, the minimum of five studies ensures the re-extraction sample remains informative when the number of included studies is small.

MANUSCRIPT CHANGES 1.7

Location: Data extraction p 9, lines 249-252

If disagreement between reviewers is identified in more than 10% of extracted fields, an additional 10% of included studies, or five studies, whichever is greater, will be extracted by both reviewers independently, and the extraction form refined before single-reviewer extraction resumes.

COMMENT 1.8 A brief Discussion section would be welcome. Is a brief synthesis of the last systematic review on this topic worth citing, or a comment outlining that there is no such publication? The literature could be very disparate. What challenges in synthesis are anticipated (they are mentioned throughout but that could be consolidated here), and what data management strategies will be considered (or are anticipated)?

RESPONSE 1.8

We agree that consolidating these points in one place strengthens the protocol. We have added a brief Discussion section covering the gap in the literature, the anticipated synthesis challenges, and our default synthesis and data management strategies.

MANUSCRIPT CHANGES 1.8

Location: Discussion p 11-12, lines 324-340

To our knowledge, no recent systematic review has synthesised longitudinal evidence on the association between infections and subsequent frailty across pathogens in adults. Existing reviews have addressed cross-sectional studies of herpesvirus seropositivity, where cytomegalovirus but no other herpesviruses was associated with frailty;[32] prevalence of frailty in people with HIV, without an uninfected comparator;[33] or

the reverse direction of association (frailty as a risk factor for infection outcomes). This review will therefore address an important gap in the evidence base.

We anticipate several challenges in synthesising the identified literature. Included studies are likely to differ in infection exposures (from single pathogens to broad measures of infection burden), frailty measurement instruments, populations, follow-up duration, and the timing of frailty assessment relative to infection. Residual confounding is likely to remain even in well-adjusted analyses, and combining different observational designs will introduce further heterogeneity in how confounders are handled. To manage these challenges, we will use structured narrative synthesis as the default and reserve meta-analysis for subsets of studies sufficiently homogeneous to permit pooling. Data management will follow a pre-specified extraction form piloted on a subset of included studies.

References:

32. Carvalho ACA, Mendes MLT, Santos VS, et al. Association between human herpes virus seropositivity and frailty in the elderly: A systematic review and meta-analysis. *Ageing Research Reviews* 2018;48 doi: 10.1016/j.arr.2018.10.009
33. Yamada Y, Kobayashi T, Condo A, et al. Prevalence of Frailty and Prefrailty in People With Human Immunodeficiency Virus Aged 50 or Older: A Systematic Review and Meta-Analysis. *Open Forum Infectious Diseases* 2022;9(5) doi: 10.1093/ofid/ofac129
