# Supplementary material for: Association between infections and frailty: a systematic review protocol
Source: BMJ Open. 2026 Sep 9;16(9):e117882. doi: 10.1136/bmjopen-2026-117882 (PMC13560988; doi:10.1136/bmjopen-2026-117882)
Supplement: Supplementary file 2 [file bmjopen-16-9-s001.docx]

**Supplementary Material**

The association between infections and frailty: a systematic review protocol

Authors: Aleixo P. Brunetti^[1]^, Mohammed Baothman^1^, Kwabena Asare^1^, Kathryn E. Mansfield^[2]^, Charlotte Warren-Gash^1^

[Supplementary table 1 - PRISMA-P 2015 Checklist	2](#_Toc221013065)

[Supplementary table 2 - Search strategy	5](#_Toc221013066)

## ****Supplementary table 1 - PRISMA-P 2015 Checklist****

**This checklist has been adapted for use with protocol submissions to *Systematic Reviews* from Table 3 in Moher D et al:** Preferred reporting items for systematic review and meta-analysis protocols (PRISMA-P) 2015 statement. *Systematic Reviews* 2015 **4**:1

| **Section/topic** | **#** | **Checklist item** | **Information reported** | | **Line number(s)** |
| --- | --- | --- | --- | --- | --- |
|  |  |  | **Yes** | **No** |  |
| **ADMINISTRATIVE INFORMATION** | | | | | |
| **Title** | | | | | |
| Identification | 1a | Identify the report as a protocol of a systematic review | ☒ | ☐ | 1 |
| Update | 1b | If the protocol is for an update of a previous systematic review, identify as such | ☒ | ☐ | 1 |
| **Registration** | 2 | If registered, provide the name of the registry (e.g., PROSPERO) and registration number in the Abstract | ☒ | ☐ | 1 |
| **Authors** | | | | | |
| Contact | 3a | Provide name, institutional affiliation, and e-mail address of all protocol authors; provide physical mailing address of corresponding author | ☒ | ☐ | 1 |
| Contributions | 3b | Describe contributions of protocol authors and identify the guarantor of the review | ☒ | ☐ | 11 |
| **Amendments** | 4 | If the protocol represents an amendment of a previously completed or published protocol, identify as such and list changes; otherwise, state plan for documenting important protocol amendments | ☐ | ☒ | N.A. |
| **Support** | | | | | |
| Sources | 5a | Indicate sources of financial or other support for the review | ☒ | ☐ | 11 |
| Sponsor | 5b | Provide name for the review funder and/or sponsor | ☒ | ☐ | 11 |
| Role of sponsor/funder | 5c | Describe roles of funder(s), sponsor(s), and/or institution(s), if any, in developing the protocol | ☐ | ☒ | N.A. |
| **INTRODUCTION** | | | | | |
| **Rationale** | 6 | Describe the rationale for the review in the context of what is already known | ☒ | ☐ | 3-4 |
| **Objectives** | 7 | Provide an explicit statement of the question(s) the review will address with reference to participants, interventions, comparators, and outcomes (PICO) | ☒ | ☐ | 4 |
| **METHODS** | | | | | |
| **Eligibility criteria** | 8 | Specify the study characteristics (e.g., PICO, study design, setting, time frame) and report characteristics (e.g., years considered, language, publication status) to be used as criteria for eligibility for the review | ☒ | ☐ | 5 |
| **Information sources** | 9 | Describe all intended information sources (e.g., electronic databases, contact with study authors, trial registers, or other grey literature sources) with planned dates of coverage | ☒ | ☐ | 6 |
| **Search strategy** | 10 | Present draft of search strategy to be used for at least one electronic database, including planned limits, such that it could be repeated | ☒ | ☐ | 6-7 |
| ***STUDY RECORDS*** | | | | | |
| Data management | 11a | Describe the mechanism(s) that will be used to manage records and data throughout the review | ☒ | ☐ | 7 |
| Selection process | 11b | State the process that will be used for selecting studies (e.g., two independent reviewers) through each phase of the review (i.e., screening, eligibility, and inclusion in meta-analysis) | ☒ | ☐ | 7 |
| Data collection process | 11c | Describe planned method of extracting data from reports (e.g., piloting forms, done independently, in duplicate), any processes for obtaining and confirming data from investigators | ☒ | ☐ | 8-9 |
| **Data items** | 12 | List and define all variables for which data will be sought (e.g., PICO items, funding sources), any pre-planned data assumptions and simplifications | ☒ | ☐ | 9 |
| **Outcomes and prioritization** | 13 | List and define all outcomes for which data will be sought, including prioritization of main and additional outcomes, with rationale | ☒ | ☐ | 6 |
| **Risk of bias in individual studies** | 14 | Describe anticipated methods for assessing risk of bias of individual studies, including whether this will be done at the outcome or study level, or both; state how this information will be used in data synthesis | ☒ | ☐ | 8 |
| ***DATA*** | | | | | |
| **Synthesis** | 15a | Describe criteria under which study data will be quantitatively synthesized | ☒ | ☐ | 9 |
|  | 15b | If data are appropriate for quantitative synthesis, describe planned summary measures, methods of handling data, and methods of combining data from studies, including any planned exploration of consistency (e.g., *I* ^2^, Kendall’s tau) | ☒ | ☐ | 10 |
|  | 15c | Describe any proposed additional analyses (e.g., sensitivity or subgroup analyses, meta-regression) | ☒ | ☐ | 10 |
|  | 15d | If quantitative synthesis is not appropriate, describe the type of summary planned | ☒ | ☐ | 9 |
| **Meta-bias(es)** | 16 | Specify any planned assessment of meta-bias(es) (e.g., publication bias across studies, selective reporting within studies) | ☒ | ☐ | 10 |
| **Confidence in cumulative evidence** | 17 | Describe how the strength of the body of evidence will be assessed (e.g., GRADE) | ☒ | ☐ | 8 |

N.A. = not applicable

## Supplementary table 2 - Search strategy

| **Table 2.1 -** **Database, Ovid MEDLINE(R) ALL <1946 to January 16, 2026>** | |
| --- | --- |
| 1 | exp frailty/ |
| 2 | Frail Elderly/ |
| 3 | frailty index.ti,ab. |
| 4 | frail*.ti,ab. |
| 5 | frailty phenotype.ti,ab. |
| 6 | (accumulation adj2 deficits).ti,ab. |
| 7 | 1 or 2 or 3 or 4 or 5 or 6 |
| 8 | exp infections/ |
| 9 | infectio*.ti,ab. |
| 10 | chronic infection*.ab,ti. |
| 11 | acute infection*.ab,ti. |
| 12 | exp respiratory infections/ |
| 13 | exp Cardiovascular Infections/ |
| 14 | exp Central Nervous System Infections/ |
| 15 | Gastrointestinal Diseases, Infectious.ti,ab. |
| 16 | Chlamydia Infections/ or Urinary Tract Infections/ or Gonorrhea/ or Urogenital Infections.mp. or Chlamydia trachomatis/ |
| 17 | Skin Diseases, Infectious/ |
| 18 | (Cellulitis or Soft Tissue Infections).mp. or Abscess.ti,ab. [mp=title, book title, abstract, original title, name of substance word, subject heading word, floating sub-heading word, keyword heading word, organism supplementary concept word, protocol supplementary concept word, rare disease supplementary concept word, unique identifier, synonyms, population supplementary concept word, anatomy supplementary concept word] |
| 19 | exp Bone Diseases, Infectious/ |
| 20 | exp Eye Infections/ |
| 21 | Otitis Media with Effusion/ or Ear Diseases, Infectious.mp. or Otitis Media/ |
| 22 | HIV Infections/ or Cholecystitis/ or Myiasis/ or Lymphadenitis/ |
| 23 | Eye Infections, Parasitic/ or Brain Abscess/ or Eye Infections, Fungal/ or Paranasal Sinus Diseases, Infectious.mp. or Meningitis, Pneumococcal/ or Sinusitis/ |
| 24 | Sepsis/ |
| 25 | Bacteremia.mp. or Bacteremia/ |
| 26 | exp Human T-lymphotropic virus 1/ |
| 27 | Herpes Simplex/ or Herpes Zoster/ or Herpes Genitalis/ or Herpes Simplex Virus Type 2.mp. or Simplexvirus/ or Herpesvirus 1, Human/ |
| 28 | Varicella-Zoster Virus.mp. or Herpesvirus 3, Human/ |
| 29 | Herpesvirus 4, Human/ or Epstein-Barr Virus.mp. |
| 30 | Cytomegalovirus.mp. or Cytomegalovirus/ or Cytomegalovirus Retinitis/ or Cytomegalovirus Infections/ |
| 31 | Roseolovirus Infections/ or Roseolovirus/ |
| 32 | HHV-6.mp. or Herpesvirus 6, Human/ |
| 33 | Herpesvirus 7, Human/ or HHV-7.mp. |
| 34 | Kaposi Sarcoma-Associated Herpesvirus.mp. or Herpesvirus 8, Human/ |
| 35 | Hepatitis B virus/ or Hepatitis B virus.mp. |
| 36 | HCV.mp. or Hepatitis C/ or Hepatitis C, Chronic/ |
| 37 | Human papillomavirus 16/ or HPV-16.mp. |
| 38 | Human papillomavirus 18/ or HPV-18.mp. |
| 39 | Polyomavirus Infections/ or DNA, Viral/ or BK Virus/ |
| 40 | JC Virus/ |
| 41 | Polyomavirus Infections/ or Merkel cell polyomavirus/ |
| 42 | exp Helicobacter pylori/ |
| 43 | exp Toxoplasma/ |
| 44 | exp Virus Diseases/ |
| 45 | exp Bacterial Infections/ |
| 46 | exp Parasitic Diseases/ |
| 47 | 8 or 9 or 10 or 11 or 12 or 13 or 14 or 15 or 16 or 17 or 18 or 19 or 20 or 21 or 22 or 23 or 24 or 25 or 26 or 27 or 28 or 29 or 30 or 31 or 32 or 33 or 34 or 35 or 36 or 37 or 38 or 39 or 40 or 41 or 42 or 43 or 44 or 45 or 46 |
| 48 | 7 and 47 |
| 49 | limit 48 to yr="2001 -Current" |

|  | **Table 2.2 - Database, Embase <1974 to 2026 January 15>** | |
| --- | --- | --- |
|  | 1 | exp frailty/ |
|  | 2 | Frail Elderly/ |
|  | 3 | frailty index.ti,ab. |
|  | 4 | frail*.ti,ab. |
|  | 5 | frailty phenotype.ti,ab. |
|  | 6 | (accumulation adj2 deficits).ti,ab. |
|  | 7 | 1 or 2 or 3 or 4 or 5 or 6 |
|  | 8 | infectio*.ti,ab. |
|  | 9 | infectious disease*.ti,ab. |
|  | 10 | pneumonia.ti,ab. |
|  | 11 | (respiratory adj3 infection*).ti,ab. |
|  | 12 | (urinary adj3 infection*).ti,ab. |
|  | 13 | skin infection.ti,ab. |
|  | 14 | (soft tissue adj3 infection*).ti,ab. |
|  | 15 | hiv infection.ti,ab. |
|  | 16 | HHV-6.ti,ab. |
|  | 17 | HHV-7.ti,ab. |
|  | 18 | Kaposi Sarcoma-Associated Herpesvirus.ti,ab. |
|  | 19 | Hepatitis B virus.ti,ab. |
|  | 20 | HCV.ti,ab. |
|  | 21 | HPV-16.ti,ab. |
|  | 22 | HPV-18.mp. |
|  | 23 | Polyomavirus Infections.ti,ab. |
|  | 24 | JC Virus.ti,ab. |
|  | 25 | Merkel cell polyomavirus.ti,ab. |
|  | 26 | Helicobacter pylori.ti,ab. |
|  | 27 | Toxoplasma.ti,ab. |
|  | 28 | 8 or 9 or 10 or 11 or 12 or 13 or 14 or 15 or 16 or 17 or 18 or 19 or 20 or 21 or 22 or 23 or 24 or 25 or 26 or 27 |
|  | 29 | 7 and 28 |
|  | 30 | limit 29 to yr="2001 -Current" |
| **Table 2.3 - Database, Global Health <1910 to 2026 Week 02>** | | |
| 1 | | exp frailty/ |
| 2 | | Frail Elderly/ |
| 3 | | frailty index.ti,ab. |
| 4 | | frail*.ti,ab. |
| 5 | | frailty phenotype.ti,ab. |
| 6 | | (accumulation adj2 deficits).ti,ab. |
| 7 | | 1 or 2 or 3 or 4 or 5 or 6 |
| 8 | | infectio*.ti,ab. |
| 9 | | infectious disease*.ti,ab. |
| 10 | | pneumonia.ti,ab. |
| 11 | | (respiratory adj3 infection*).ti,ab. |
| 12 | | (urinary adj3 infection*).ti,ab. |
| 13 | | skin infection.ti,ab. |
| 14 | | (soft tissue adj3 infection*).ti,ab. |
| 15 | | hiv infection.ti,ab. |
| 16 | | HHV-6.ti,ab. |
| 17 | | HHV-7.ti,ab. |
| 18 | | Kaposi Sarcoma-Associated Herpesvirus.ti,ab. |
| 19 | | Hepatitis B virus.ti,ab. |
| 20 | | HCV.ti,ab. |
| 21 | | HPV-16.ti,ab. |
| 22 | | HPV-18.mp. |
| 23 | | Polyomavirus Infections.ti,ab. |
| 24 | | JC Virus.ti,ab. |
| 25 | | Merkel cell polyomavirus.ti,ab. |
| 26 | | Helicobacter pylori.ti,ab. |
| 27 | | Toxoplasma.ti,ab. |
| 28 | | 8 or 9 or 10 or 11 or 12 or 13 or 14 or 15 or 16 or 17 or 18 or 19 or 20 or 21 or 22 or 23 or 24 or 25 or 26 or 27 |
| 29 | | 7 and 28 |
| 30 | | limit 29 to yr="2001 -Current" |

1. Faculty of Epidemiology and Population Health, London School of Hygiene and Tropical Medicine, London, United Kingdom
2. School of Health and Care Sciences, University of Lincoln, Leicester, United Kingdom.
